# Interdisciplinary Research in Artificial Intelligence: Challenges and Opportunities

**DOI:** 10.3389/fdata.2020.577974

**Published:** 2020-11-23

**Authors:** Remy Kusters, Dusan Misevic, Hugues Berry, Antoine Cully, Yann Le Cunff, Loic Dandoy, Natalia Díaz-Rodríguez, Marion Ficher, Jonathan Grizou, Alice Othmani, Themis Palpanas, Matthieu Komorowski, Patrick Loiseau, Clément Moulin Frier, Santino Nanini, Daniele Quercia, Michele Sebag, Françoise Soulié Fogelman, Sofiane Taleb, Liubov Tupikina, Vaibhav Sahu, Jill-Jênn Vie, Fatima Wehbi

**Affiliations:** ^1^ INSERM U1284, Université de Paris, Center for Research and Interdisciplinarity (CRI), Paris, France; ^2^ Inria, Villeurbanne, France; ^3^ Imperial College London, London, United Kingdom; ^4^ University of Rennes, Rennes, France; ^5^ Inria Flowers, Paris and Bordeaux, France; ^6^ ENSTA Paris, Institut Polytechnique Paris, Paris, France; ^7^ Université Paris-Est, LISSI, Vitry sur Seine, France; ^8^ Université de Paris, France and French University Institute (IUF), Paris, France; ^9^ Université Grenoble Alpes, Inria, CNRS, Grenoble INP, LIG, Grenoble, France; ^10^ Nokia Bell Labs, Cambridge, United Kingdom; ^11^ TAU, LRI-CNRS–INRIA, Universite Paris-Saclay, France; ^12^ Hub France Intelligence Artificielle, Paris, France; ^13^ Nokia Bell Labs, Paris, France; ^14^ Inria, Lille, France

**Keywords:** artificial intelligence, interdisciplinary science, education, ethics, auditability, interpretability

## Abstract

The use of artificial intelligence (AI) in a variety of research fields is speeding up multiple digital revolutions, from shifting paradigms in healthcare, precision medicine and wearable sensing, to public services and education offered to the masses around the world, to future cities made optimally efficient by autonomous driving. When a revolution happens, the consequences are not obvious straight away, and to date, there is no uniformly adapted framework to guide AI research to ensure a sustainable societal transition. To answer this need, here we analyze three key challenges to interdisciplinary AI research, and deliver three broad conclusions: 1) future development of AI should not only impact other scientific domains but should also take inspiration and benefit from other fields of science, 2) AI research must be accompanied by decision explainability, dataset bias transparency as well as development of evaluation methodologies and creation of regulatory agencies to ensure responsibility, and 3) AI education should receive more attention, efforts and innovation from the educational and scientific communities. Our analysis is of interest not only to AI practitioners but also to other researchers and the general public as it offers ways to guide the emerging collaborations and interactions toward the most fruitful outcomes.

## Introduction

Artificial Intelligence (AI), which typically refers to the artificial creation of human-like intelligence that can learn, perceive and process information, is rapidly becoming a powerful tool for solving image recognition, document classification (Vapkin, 1995; LeCun et al., 2015) as well as for the advancement of interdisciplinary problems. It is often considered to be a powerful computational tool that can be applied to many complex problems which have not been successfully addressed so far. However, this is not a one way street, other fields such as neuroscience (Hassabis et al., 2017; Ullman, 2019), developmental psychology (Bennetot et al., 2020; Charisi et al., 2020), developmental robotics (Oudeyer, 2011; Moulin-Frier and Oudeyer, 2013; Doncieux et al., 2020) and evolutionary biology (Gobeyn et al., 2019) can inspire AI research itself, for example by suggesting novel ways to structure data (Timmis and Knight, 2002), or helping discover new algorithms, such as neural networks, which are inspired from the brain (Rosenblatt, 1958). Of course, combining AI with other fields is not without challenges. Like any time when fields synergize, barriers in communication arise, due to differences in terminologies, methods, cultures, and interests. How to bridge such gaps remains an open question, but having a solid education in both machine learning and the field of interest is clearly imperative. An example of cross-pollination interdisciplinary program showing the success of these approaches is not utopic is Frontier Development Lab, a cooperative agreement between NASA, the Seti Institute, and ESA set up to work on AI research for space science, exploration and all humankind (Frontier Development Lab). Besides multidisciplinarity, advocating for ethics and diversity (Agarwal et al., 2020) is a must to account for biased models (Hendricks et al., 2018; Denton et al., 2019) and avoid stereotypes being perpetuated by AI systems (Gebru, 2019). For instance, interdisciplinary approaches, e.g., including art and science, as well as ensuring minorities are well represented among both the users and the evaluators of the latest eXplainable AI techniques (Arrieta et al., 2020), can make AI more accessible and inclusive to otherwise unreachable communities.

While the AI revolution in research, healthcare and industry is presently happening at full speed, its long term impact on society will not reveal itself straight away. In research and healthcare, this might lead to blindly applying AI methods to problems for which, to date, the technology is not ready [e.g., IBM’s Watson for oncology (Strickland, 2019)], and to ethically questionable applications [e.g., predicting sexual orientations from people's faces (Wang and Kosinski, 2017), using facial recognition in law enforcement or for commercial use (Clearview)]. AI can be used as a tool to improve data privacy (e.g., for deidentification, www.d-id.com) or for threat identification, but it is more often seen as itself being a threat to IT systems (Berghoff et al., 2020), e.g., in the cases of biometric security and privacy (Jiang et al., 2017). AI can be a target of attacks with vulnerabilities qualitatively new to AI systems [e.g. adversarial attacks and poisoning attacks (Qiu et al., 2019)] as well as a powerful new tool used by the attackers (Dixon and Eagan, 2019). In industry, AI chatbots ended up being racist, reflecting the training data that was presented to the algorithm, recruitment software ended up being gender-biased; and risk assessment tools developed by a US contractor sent innocent people to jail (Dressel and Farid, 2018). A more careful consideration of the impact of AI is clearly needed by following global and local ethics guidelines for trustworthy (Smuha, 2019) and responsible AI (Arrieta et al., 2020).

While a large number of industries have seen a potential in this technology and invested colossal amounts of money to incorporate AI solutions in their businesses, predictions made by AI algorithms can be frightening and without a proper educational framework, lead to a societal distrust. In this opinion paper we put forward three research topics that we believe AI research should accentuate on,

1. How can an interdisciplinary approach towards AI benefit from and contribute to the AI revolution? While AI is already used in various scientific fields, it should go beyond solely predicting outcomes towards conducting exploratory analysis and finding new patterns in complex systems. Additionally, in the future development of AI, the reverse direction should also be considered, namely investigating ways in which AI can take inspiration and can benefit from other fields of science.
2. How could regulatory agencies help correct existing data biases and discriminations induced by AI? To ensure this, AI research must be accompanied by decision explainability and dataset and algorithm bias analysis as well as creation of regulatory agencies and development of evaluation methodologies and tools. In all cases, AI research should guarantee privacy as well as economical and ecological sustainability of the data and algorithms based on it.
3. How can we manage the impact of this AI revolution once AI tools are deployed in the real world, particularly how to ensure trust of the scientific peers and the general public? This includes establishing public trust in AI through education, explainable solutions, and regulation.

By considering these three aspects, interdisciplinary research will go beyond the considerations of individual disciplines to take broader and more thoughtful views of the promised digital revolutions. Our recommendations are a result of in-person discussions within a diverse group of researchers, educators, and students, during a 3-day thematic workshop, which has been collectively written and edited during and after the meeting. While not comprehensive, we believe they capture a broad range of opinions from multiple stakeholders and synthesize a feasible way forward.

## Part I: Artificial Intelligence and Interdisciplinary Research

The relationship between AI and interdisciplinary research must be considered as a two-way street. While one direction may be more well known (applying AI to other fields), here we consider both directions: 1) from AI to other fields and 2) from other fields to AI. Then we argue that applying knowledge from other fields to AI development is equally important in order to move forward and to achieve the full potential of the AI revolution.

### From Artificial Intelligence to Other Fields

Using AI to make predictions or decisions in e.g. quantitative science, healthcare, biology, economy and finance has been extensively, and possibly excessively done over the past several years. While the application of AI to these domains remains an active area of research, we believe that the biggest challenge for the future of AI lies ahead. Rather than just predicting or making decisions, AI solutions should be developed to conduct exploratory analyses, i.e., to find new, interesting patterns in complex systems or facilitate scientific discovery (Raghu and Schmidt, 2020). Specific cases where this direction has already been explored include e.g., drug discovery (Vamathevan et al., 2019), the discovery of new material (Butler et al., 2018), symbolic math (Lample and Charton, 2019; Stanley et al., 2019) or the discovery of new physical laws (Both et al., 2019; Iten et al., 2020; Udrescu and Tegmark, 2020). *Will AI succeed in assisting humans in the discovery of new scientific knowledge? If so, in which domain will it happen first? How do we speed up the development of new AI methods that could reach such goals?* These are some questions that should inspire and drive the applications of AI in other fields.

Another possible approach consists of using AI models as experimental “guinea pigs” for hypothesis testing. In the domain of neuroscience, one standard methodology consists of analyzing which AI model is best at predicting behavioral data (from animals or humans) in order to support or inform hypotheses on the structure and on the function of biological cognitive systems (Gauthier and Levy, 2019). In that case, the process of training the AI-agent is an experiment in itself since the intrinsic interest does not lie in the performance of the underlying algorithm per se but instead in its ability to explain cognitive functions. *Can we create an AI algorithm that will replace all stages of scientific process, from coming up with questions, generating the data, to analysis and interpretation of results?* Such automated discovery is considered as the ultimate goal by some experts, but so far remains out of reach (Bohannon, 2017).

### From Other Fields to Artificial Intelligence

Whereas AI approaches are readily impacting many scientific fields, those approaches also continue to benefit from insights from fields such as neuroscience (Hassabis et al., 2017; Samek et al., 2019; Ullman, 2019; Parde et al., 2020), for example the similarities between machine and human-like facial recognition (Grossman et al., 2019) and the use of the face space concept in deep convolutional neural networks (O’Toole et al., 2018; Parde et al., 2020). Other fields impacting AI research include evolutionary biology (Gobeyn et al., 2019) and even quantum mechanics (Biamonte et al., 2017). One of the biggest successes of integrating insights from other fields in modern day AI, the perceptron, became the prelude to the modern neural networks of today (Rosenblatt, 1958). Perceptrons and neural networks can be considered analogous to a highly reduced model of cortical neural circuitry. Other examples are algorithms such as reinforcement learning which drew inspiration from principles of developmental psychology from the 50s (Skinner, 2019) and have been influencing the field of developmental robotics (Cangelosi and Schlesinger, 2015) since the 2010s. Further illustration of this cross-fertilization can be seen in bio-inspired approaches, where principles from natural systems are used to design better AI, e.g., neuroevolution algorithms that evolve neural networks through evolutionary algorithms (Floreano et al., 2008). Finally, the rise of quantum computers and quantum-like algorithms could further expand the hardware and algorithmic toolbox for AI (Biamonte et al., 2017). Despite these important advances in the last decade, AI systems are still far from being comparable to human intelligence (and to some extent to animal intelligence), and several questions remain open. For instance, *how can an AI system learn and generalize while being exposed to only a small amount of data? How to bridge the gap between low-level neural mechanisms and higher-level symbolic reasoning?*

While AI algorithms are still mostly focused on the modeling of purely cognitive processes (e.g., learning, abstraction, planning…), a complementary approach could consider intelligence as an emergent property of cognitive systems through their coupling with environmental, morphological, sensorimotor, developmental, social, cultural and evolutionary processes. In this case, the highly complex dynamic of the ecological environment is driving the cognitive agents to continuously improve in an ever-changing world, in order to survive and to reproduce (Pfeifer and Bongard, 2006; Kaplan and Oudeyer, 2009). This approach draws inspiration from multiple scientific fields such as evolutionary biology, developmental science, anthropology or behavioral ecology. Recent advances in reinforcement learning have made a few steps in this direction. Agents capable of autonomously splitting a complex task into simpler ones (auto-curriculum) can evolve more complex behaviors through coadaptation in mixed cooperative-competitive environments (Lowe et al., 2017). In parallel, progress has also been made in curiosity-driven multi-goal reinforcement learning algorithms, enabling agents to autonomously discover and learn multiple tasks of increasing complexity (Doncieux et al., 2018). Finally, recent work has proposed to jointly generate increasingly complex and diverse learning environments and their solutions as a way to achieve open-ended learning (Doncieux et al., 2018). One related research direction are studies of systems that sequentially and continually learn (Lesort et al., 2020) in a lifelong setting, i.e., continual learning without experiencing the well known phenomenon of catastrophic forgetting (Traoré et al., 2019). When combined, this research puts forward the following questions: *How can we leverage recent advances that situate AI agents within realistic ecological systems? How does the dynamic of such systems drive the acquisition of increasingly complex skills?*

## Part II: Artificial Intelligence and Society

The rise of AI in interdisciplinary science brings along significant challenges. From biased hiring algorithms, to deep fakes, the field has struggled to accommodate a rapid growth and an increasing complexity of algorithms (Chesney and Citron, 2019). Moreover, the lack of explainability (Arrieta et al., 2020) has slowed down its impact in areas such as quantitative research and prevents the community to further develop reproducible and deterministic protocols. Here we propose methodologies and rules to mitigate the inherent risks that arise from applying complex and non-deterministic AI methods. In particular we discuss how general scientific methodologies can be adapted for AI research and how auditability, interpretability and environmental neutrality of results can be ensured.

### Adapting the Scientific Method to Artificial Intelligence-Driven Research

To ensure that AI solutions perform as we intended, it is important to clearly formulate the problem and to state the underlying hypothesis of the model. By matching formal problem expression/definitions to laws (intentions), functional and technical specifications, we ensure that the project has a well established scope and a path towards achieving this goal. These specifications have been set forward by the GDPR (General Data Protection Regulation) that published a self assessment template guiding scientists and practitioners to prepare their AI projects for society (Bieker et al., 2016). In short, products and services resulting from AI decision making must clearly define their applicability and limitations. Note that this differs from problem definition since it involves explicitly stating how the algorithm will address part or all of the original problem. The developers have to explicitly detail how they handle extreme cases and show that security of the user is ensured. It should be mandatory for the owner and user of the data to clearly and transparently state the known biases expressed by the dataset (similar to the way the secondary effects of medicines are clearly stated on the medication guide). While some of these are already addressed by the GDPR in the EU, similar regulation and standards are needed globally. An alternative, complementary approach would be to rely on the classical scientific method practices developed over the centuries. Relying on observation, hypothesis formulation, experimentation (Rawal et al., 2020) and evaluation allows us to understand causal relationships and promotes rigorous practices. AI would certainly benefit from explicitly integrating these practices into its research ecosystem (Forde and Paganini, 2019).

### Biases and Ethical Standards in Artificial Intelligence

To control the functioning of AI algorithms and their potential inherent biases, clear, transparent and interpretable methodologies and best practices are required. Trustworthiness of AI-driven projects can be ensured by, for example, using open protocols of the algorithms functionality, introducing traceability (logs, model versioning, data used and transformations done on data) or the pre-definition of insurance datasets. In transversal domains such as software development, tools have been devised to prevent mistakes and model deterioration over time (such as automated unit tests). Establishing similar standards for AI would force data scientists to design ways to detect and eliminate biases, ultimately making sure that the algorithm is behaving as intended. If ethical standards can be encoded in the algorithm, then regulation can be imposed on the optimized objectives of AI models (Jobin et al., 2019).

### Auditability and Interpretability

The goal of AI should be to improve human condition and not further aggravate either existing inequalities (Gebru, 2019) or environmental issues in our societies. The AI service and product developers are likely to be at the center of this challenge - they are the ones that can directly prevent errors and biases in input data or future applications. They present a priori knowledge that can lead to or prevent misuse (conscious or unconscious). It is tempting to extensively employ libraries and “ready-to-use” code samples, as these make the production process faster and easier. However, especially when used by non-experts, the key features of AI models, e.g., data recasting, could easily be implemented incorrectly. The secondary users of AI tools must be able to measure the biases of their input data and obtained results, which can be done only if they are both aware of potential problems and if they have the necessary tools readily available.

As with any software, failures and mistakes will inevitably arise and a system has to be in place to assess how AI tools and services behave not only during development but also “in production.” The combination of decision logs and model versioning can allow us to verify and ensure the product outcomes are the ones intended. Here the question of independent authorities comes in order to regularly audit the AI products around us. Companies and AI product developers must be capable of “opening the black box” and clearly exposing the monitoring they perform over an algorithm. Opening the black box has already been set as an important goal in AI research (Castelvecchi, 2016), even if not all experts agree that this is necessary (Holm, 2019). It includes not only making the currently used model transparent, but more importantly being able to explain how it was designed, and examining its past states and decisions. For example, developers must track data drifting and deploy policies preventing an algorithm to produce unintended outcomes. So far, this has been left to good practices of individual developers, but we can envision construction of an authority in charge of auditing AI products regularly. One proposed approach has been to impose *Adversarial Fairness* during training or on the output (Adel et al., 2019). Independently of a particular way to ensure auditability and interpretability, the process should be co-designed not only by AI practitioners but all stakeholders, including the general public, following open science principles (Greshake Tzovaras et al., 2019). Auditability and interoperability considerations complement and extend the more obvious and direct requirements of robustness, security and data privacy in AI.

Finally, as for any technology, the usefulness of AI will have to be assessed against its environmental impact. In particular, life cycle assessment of AI solutions should be systematic. Here also, auditing by independent authorities could be a way to enforce environmental neutrality (Schwartz et al., 2019).

### Education Through and About Artificial Intelligence Technologies

Besides impacting research and industry directly, AI is transforming the job market at a rapid pace. It is expected that approximately 80% of the population will be affected by these technological advancements in the near future (HolonIQ). Highly complex jobs (e.g., the medical, juridical or educational domains) will be redefined, some simpler, repetitive tasks will be replaced or significantly assisted by AI and new jobs will appear in the coming decades. For instance, budget readjustment and reeducation of people who lose their jobs, towards a clean energy shift, with only about 30% coming from governments (which amounts to less than 10% of the funds committed to coronavirus economic relief), could positively shift climate change (Florini, 2011). However, workers of these different fields received little to no formal education on AI, and more initiatives on sustainable AI (such as EarthDNA ambassadors or TeachSDG Ambassadors) are needed. Therefore, the AI transformation should come along with a transformation in education where educational and training programs will have to be adapted to these different existing professions.

The transformation in education can be implemented on four different levels: academic institutions, companies and governments. Academic institutions should not only prepare AI experts by providing in-depth training to move forward AI research but also focus on interdisciplinarity and attract diversity in AI. Three main axes for AI education should be: 1) high level AI experts who can train future generations 2) AI practitioners who can raise public awareness in their research and (3), broader public that can be informed directly, leading to decrease in a priori distrust.

The end users and beneficiaries of AI services and products, as the most numerous part of the population, must play a central role in their development. It is they who should have the final say on what global use of AI technologies should be pursued. However, to do so, they must have a chance to learn the fundamental principles of AI. This is not fundamentally different from educating the general public about any scientific topic with a global societal impact, may it be medical (e.g., antibiotic resistance, vaccination) or environmental (e.g., climate change). Providing the information and training at scale is not a trivial task, due to at least two major issues: 1) the motivation of the general public and 2) the existence of appropriate educational tools. Various online resources are available targeting the general public, such as Elements of AI in Finland or *Objectif’IA* in France. Interestingly, in the case of AI, the problem itself could also be a part of a possible solution - we can envisage AI playing a central role in creating adaptive learning paths, individual-based learning programs addressing the needs and interests of each person affected by AI technology. Educational tools designed with AI can motivate each individual by providing relevant, personalized examples and do it at the necessary scale. Interactions between AI and education is yet another example of interdisciplinarity in AI (Oudeyer et al., 2016), which can directly benefit not only the two fields, education and AI, but society and productivity as a whole.

## Conclusion

AI is currently ever present in science and society, and if the trend continues, it will play a central role in the education and jobs of tomorrow. It inevitably interacts with other fields of science and in this paper we examined ways in which those interactions can lead to synergistic outcomes. We focused our recommendations on mutual benefits that can be harnessed from these interactions and emphasized the important role of interdisciplinarity in this process. AI systems have complex life cycles, including data acquisition, training, testing and deployment, ultimately demanding an interdisciplinary approach to audit and evaluate the quality and safety of these AI products or services. Furthermore in Part II we focused on how AI practitioners can prevent biases through transparency, explainability, inclusiveness and how robustness, security and data privacy can and should be ensured. Finally we emphasize the importance of education for and through AI to allow the whole society to benefit from this AI transition. We offer recommendations from the broad community gathered around the workshop resulting in this paper, with the goal of contributing, motivating and informing the conversion between AI practitioners, other scientists, and the general public. In this way, we hope this paper is another step towards harnessing the full potential of AI for good, in all its scientific and societal aspects.

## Data Availability Statement

The original contributions presented in the study are included in the article/supplementary material, further inquiries can be directed to the corresponding author/s.

## Author Contributions

RK and DM wrote the initial draft and supervised the project. All the other authors contributed equally to the conceptualization, data curation, and investigation presented in this paper.

## Conflict of Interest

Authors LT and DQ are employed by the company Nokia Bell Labs.

The remaining authors declare that the research was conducted in the absence of any commercial or financial relationships that could be construed as a potential conflict of interest.
